# Functional and morphological differences of the lung upon acute and chronic ozone exposure in mice

**DOI:** 10.1038/s41598-018-28261-9

**Published:** 2018-07-13

**Authors:** Chloé Michaudel, Louis Fauconnier, Yvon Julé, Bernhard Ryffel

**Affiliations:** 1 Laboratory of experimental and molecular immunology and neurogenetics (INEM), UMR 7355 CNRS-University of Orleans, University of Orleans, F-45071 Orleans-Cedex2, France; 2 Artimmune SAS, Orléans, France; 3 Biocellvia, Marseille, France; 4 Institute of Infectious Diseases and Molecular Medicine (IDM), Division of Immunology, University of Cape Town, Anzio Road, Observatory 7925 Cape Town, Republic of South Africa; 5 Department of Clinical Immunology, Third Hospital at Sun Yat-sen University, Guangzhou, China

## Abstract

Environmental air pollutants including ozone cause severe lung injury and aggravate respiratory diseases such as asthma and COPD. Here we compared the effect of ozone on respiratory epithelium injury, inflammation, hyperreactivity and airway remodeling in mice upon acute (1ppm, 1 h) and chronic exposure (1.5ppm, 2 h, twice weekly for 6 weeks). Acute ozone exposure caused respiratory epithelial disruption with protein leak and neutrophil recruitment in the broncho-alveolar space, leading to lung inflammation and airway hyperresponsiveness (AHR) to methacholine. All these parameters were increased upon chronic ozone exposure, including collagen deposition. The structure of the airways as assessed by automatic numerical image analysis showed significant differences: While acute ozone exposure increased bronchial and lumen circularity but decreased epithelial thickness and area, chronic ozone exposure revealed epithelial injury with reduced height, distended bronchioles, enlarged alveolar space and increased collagen deposition, indicative of peribronchiolar fibrosis and emphysema as characterized by a significant increase in the density and diameter of airspaces with decreased airspace numbers. In conclusion, morphometric numerical analysis enables an automatic and unbiased assessment of small airway remodeling. The structural changes of the small airways correlated with functional changes allowing to follow the progression from acute to chronic ozone induced respiratory pathology.

## Introduction

**Figure 1:**
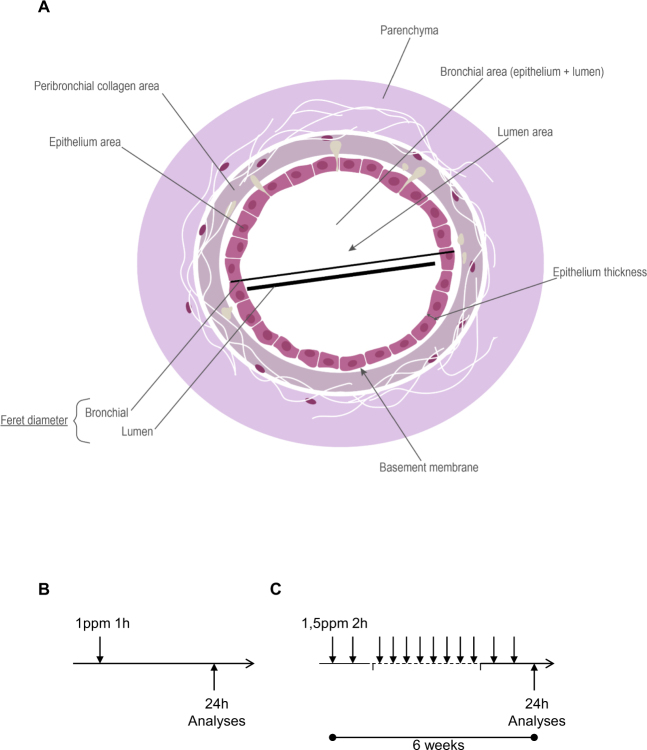
Scheme of the small airway with morphometric parameters and ozone models Schematic picture of a small airway (**A**), and experimental protocol of acute/single (**B**) and chronic/six weeks ozone exposure (**C**).

Pulmonary tissue damage is inflicted by exposure to different external factors such as air pollution, tobacco smoke, particulate matter and allergens, causing epithelial alteration, inflammation, fibrosis, emphysema and airway hyperresponsiveness (AHR). Chronic environmental exposure leads to injury and chronic inflammation such as COPD, asthma or pneumoconiosis^1–3^. Ozone is a major air pollutant, causing irritation and injury accompanied by the recruitment of immune and inflammatory cells in the lung with bronchiolar epithelial desquamation and alveolar septum disruption, resulting in emphysema and AHR^4–10^. Ozone-induced injury and inflammation is dependent upon the dose and frequency of exposure^11–13^. To quantify microscopic tissue alteration, an arbitrary quantification is routinely performed, using semi-quantitative scoring of various parameters such as cell infiltration, epithelial damage and fibrosis^14^. In order to conduct a more accurate and reliable quantitative evaluation of bronchial structural changes, we developed an automatic numerical analysis which is totally observer-independent. We focused on the analysis of two key targets: epithelial cell injury and collagen content (Fig. 1A). Analysis of bronchial epithelial damage was performed on the small bronchioles by measuring multiple morphometric parameters: the whole bronchial area, lumen area, epithelial wall area and thickness, bronchial and lumen circularity and bronchial Feret diameter. Concurrently, we developed an automatic numerical quantification of collagen content expressed in the adjacent area of the bronchial wall. To assess injury which can occur in alveolar parenchyma and lead to emphysema, a new automatic numerical quantification of the density, diameter and number of airspaces was carried out instead of the standard mean linear intercept (Lm) measurement. We report here that the data obtained by the new digital imaging approach enabled an objective, accurate and reliable morphometric evaluation of the integrity of the airways, and correlating with functional and inflammatory analyses. These combined histological and functional investigations give access to a more thorough analysis of airways remodeling, and therefore represent an efficient and reliable approach to test the efficacy of drug candidates.

## Results

### Assessment of cytokines and airway hyperreactivity (AHR)

**Figure 2:**
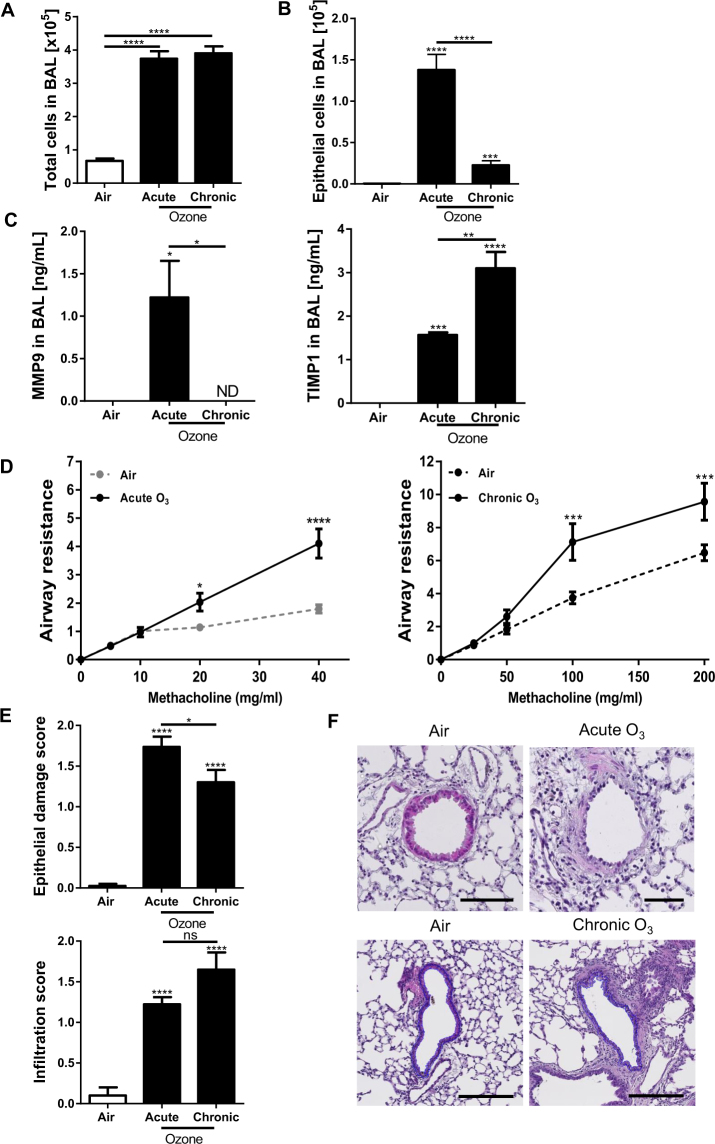
Cytokines, lung function and tissue remodeling after ozone exposure Total cell count in BAL (**A**), epithelial cell desquamation (**B**) and protein measurement of MMP-9 and TIMP-1 (**C**). Lung function measurement (**D**) and tissue remodeling: epithelial damage and infiltration score (**E**) with representative histological images of analyzed small bronchi (**F**). Data were pooled from 3 independent experiments with 5–6 mice per group. Comparison of the ozone-exposed groups with air group. *p < 0.05, **p < 0.01, ***p < 0.001, ****p < 0.0001. p-value < 0.05 was considered statistically significant. ns: non-significant. Scale bars: 100 µm.

The effect of acute and chronic ozone exposure was investigated in WT mice (Fig. 1B,C). Acute and to a lesser extent chronic ozone exposure induced a significant cell recruitment (Fig. 2A) and desquamation of EPCAM^+^ epithelial cells in BAL (Fig. 2B). MMP-9 levels in BAL were increased after acute, but not chronic ozone exposure (Fig. 2C), while TIMP1 levels were higher in BAL after chronic ozone exposure (Fig. 2C). AHR was recorded after acute or chronic ozone exposure following administration of increasing concentrations of methacholine (25–200 mg/mL). A marked increase of AHR was found in the acute ozone model in response to low doses of methacholine (20 mg/ml), while much higher doses of methacholine were required for the chronic ozone model (100 mg/ml) (Fig. 2D).

### Semi-quantitative analysis of epithelial damage and inflammation

Semi-quantitative histological analysis assessed by scoring evaluation was performed to quantify epithelial small airway damage and inflammatory cell infiltration. In both acute and chronic ozone models there was a statistically significant increase of the mean scoring value of epithelial damage and cell infiltration (Fig. 2E,F**)**. The epithelial small airway damage was visually characterized by both flattening of epithelial cells and patchy desquamation.

### Morphometric analysis of small airway remodeling

**Figure 3:**
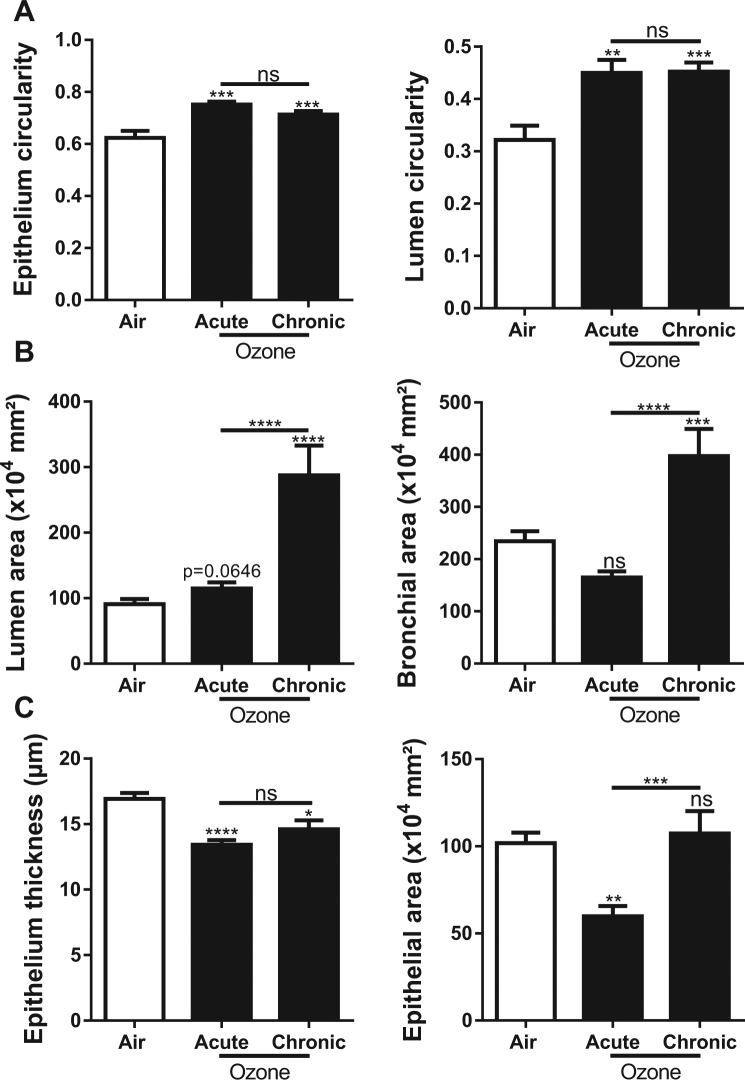
Effect of acute and chronic ozone exposure on morphometric parameters of small bronchioles. Measurement of the circularity of epithelium and lumen (**A**), the area of lumen, bronchiole and epithelium (**B**,**C**), and the thickness of epithelium (**C**). Data were pooled from 3 independent experiments with 5–6 mice per group. The circularity of epithelium was assessed automatically from the limit of its basal membrane and that of lumen from the lumen limit of the epithelial cells. The area of lumen corresponds to the difference between the area of the bronchiole and the lumen. Note that the area of the epithelium was closed and sometimes lesser than that of the lumen a feature of the small bronchi. The thickness of the bronchi was automatically assessed from hundreds of measures performed all around the epithelium. Comparison of the ozone-exposed groups with air group. *p < 0.05, **p < 0.01, ***p < 0.001. p-value < 0.05 was considered statistically significant. ns: non-significant.

The morphometric analysis was performed on small bronchi selected automatically from their Feret diameter (the largest bronchial diameter) ranged between 100 and 500 µm. These small bronchi were devoid of smooth muscle layer. In order to provide a precise and reliable quantification of structural changes, we developed a fully automatic measurement of multiple key morphometric parameters, namely, the epithelium area and thickness, the bronchiole and lumen areas, and the bronchiole and lumen circularity. Epithelium and lumen circularity are higher in bronchioles exposed to ozone both models than air group (Fig. 3A), translate epithelium damage induced by ozone. Acute ozone exposure did not induce significant changes of bronchiole and lumen area, whereas chronic ozone exposure resulted in a significant increase of both bronchiole and lumen areas (Fig. 3B,C). Furthermore, acute ozone exposure induced a significant decrease of epithelium wall area and thickness (Fig. 3C). Chronic ozone exposure did not change the epithelium area, but caused a significant decrease of epithelium thickness (Fig. 3C). It is worth noting that the decrease of epithelium area and thickness in the acute ozone model is in agreement with the structural changes that were observed, which demonstrated the flattening and patchy desquamation of epithelial cells as quantified by the scoring evaluation (Fig. 2E,F). Structural changes of bronchial epithelium were also evidenced by the significant increase of epithelium and lumen circularity in both the acute and chronic ozone groups. The increase in bronchial circularity indicates that ozone induced significant changes in the shape of the bronchioles which were markedly more round.

### Ozone-induced lung fibrosis

**Figure 4:**
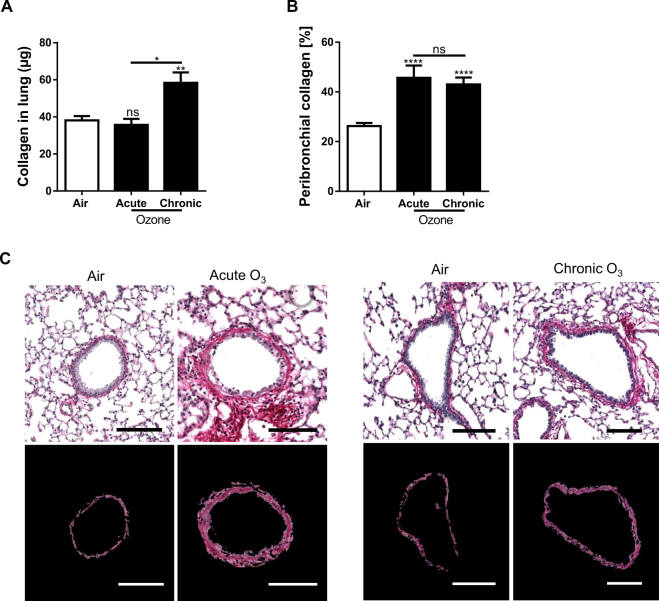
Effect of ozone exposure on collagen production Quantification of collagen content in air and ozone-exposed groups by means of Sircol™ assay method (**A**) and digital image analysis (**B**). Sircol™ assay allows to measure the collagen content expressed in the whole lung whereas digital image analysis the collagen content expressed in the vicinity of the small bronchioles (peribronchial collagen). It is noteworthy to observe that only image analysis allowed to detect a significant increase of collagen content in the acute ozone group. Representative images of air and ozone groups show the discrimination of collagen content around small bronchi which allows to access to its quantification (**C**). Data (mean ± SEM) were pooled from 3 independent experiments with 5–6 mice per group. Comparison of the ozone-exposed groups with air group. **p < 0.01, ****p < 0.0001. p-value < 0.05 was considered statistically significant. ns: non-significant. Scale bars: 100 µm.

Collagen content was assessed in acute and chronic ozone models in the lung homogenate biochemically using the Sircol assay™. Collagen was increased in the lung only upon chronic ozone exposure, but not after a single exposure (Fig. 4A). Microscopic analysis of lung tissues revealed collagen deposition in peribronchiolar area by picrosirius red staining, which was quantified using our dedicated software image analysis. Interestingly, we found a significant increase of peribronchial collagen content in both acute and chronic ozone models (Fig. 4B,C). It is worth noting that the increase of peribronchial collagen content induced by chronic ozone exposure was correlated with the increase of collagen content in the whole pulmonary lobe as assessed by the Sircol assay.

### Progression to emphysema

**Figure 5:**
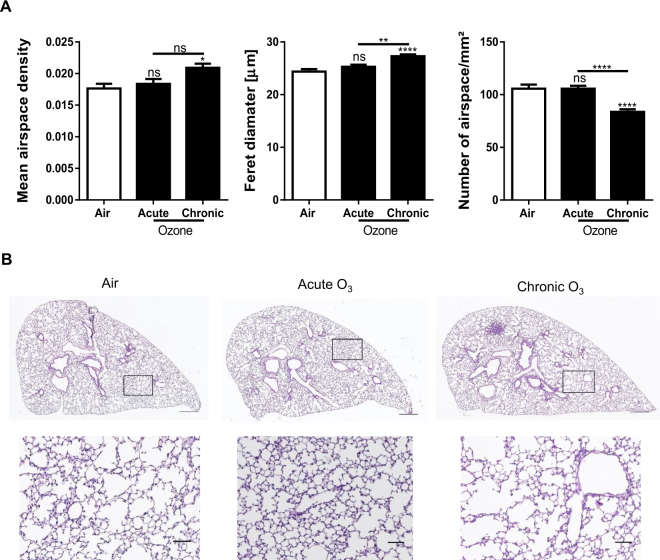
Progression from acute to chronic inflammation with emphysema. Measurement ofairspace density, Feret diameter and number of airspaces in WT mice exposed to acute and chronic ozone (**A**), and representative histological images of air, acute and chronic ozone groups (**B**). Data (mean ± SEM) were pooled from 2 independent experiments with 5–6 mice per group. Note in B the presence of emphysema in the chronic ozone group which was evidenced by a significant increase of airspace density, airspace diameter and in contrary by a significant decrease of the airspace number. Calibrations: upper images, 500 µm; lower images, 75 µm. Comparison of the ozone-exposed groups with air group. **p < 0.01, ****p < 0.0001. p-value < 0.05 was considered statistically significant. ns: non-significant.

In order to detect the presence of emphysema in the acute and chronic ozone exposure groups, we measured changes in airspace density, airspace diameter and the number of airspaces per mm^2^ of parenchymal tissue (Fig. 5). In the chronic ozone exposure group, a significant increase was observed in airspace density and airspace diameter, but a decrease in the number of airspaces. In the acute ozone group, no significant change was found in all morphometric parameters.

## Discussion

**Figure 6:**
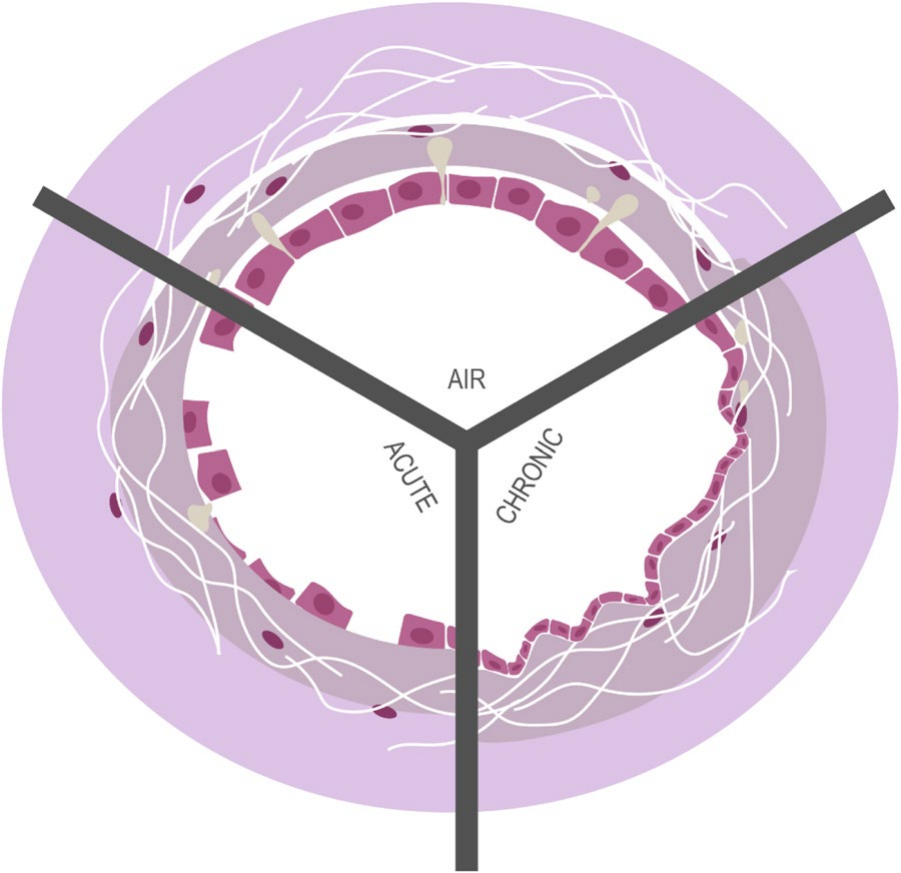
Schematic differences between acute and chronic lung changes after acute and chronic ozone exposure. Without ozone exposure, the epithelial barrier is normal with regular epithelial cells and a very thin collagen layer. Acute ozone exposure causes severe lung damage, with disruption of tight junctions, epithelial desquamation and peribronchial collagen deposition indicating imminent fibrosis. Following chronic exposure, the lumen and bronchial areas are increased with no modification of epithelium area, but accompanied by a reduction in epithelium thickness, indicative of stretching of the epithelium wall typical for emphysema associated with fibrosis.

The effect of ozone exposure on the airways is not yet well elucidated. We reported that a single exposure causes an acute disruption of the respiratory barrier with the desquamation of necrotic epithelial cells, protein leak and neutrophilic inflammation^15^. There has been no direct comparison between acute and chronic ozone lung pathologies. Here we compared the functional and morphologic changes of acute and chronic ozone exposure as schematically summarized in Fig. 6.

The fully automatic analysis we developed in the present study eliminates intra- and inter-variations in morphometric measurements as imposed by the experimenters, as well as by any manual manipulations implemented in the course of the image analysis workflow. Moreover, the computer numerical analysis we conducted provides multiple new key morphometric parameters which allowed a deeper analysis of morphometric changes occurring in the remodeling of lung tissue following acute and chronic exposure to ozone.

We found that acute ozone exposure induced significant structural changes in bronchiolar epithelium, characterized by both a significant increase of epithelial cells in BAL and semi-quantitative score of epithelial damage. These results are in accordance with a significant decrease of the epithelium thickness and area measured in acute ozone-exposed lung sections. Nevertheless, these structural changes after acute ozone exposure did not modify significantly the area of bronchiole and lumen, unlike their circularity. The increase of bronchiole and lumen circularity is the result of morphological changes observed in part in epithelial cells and very likely in the peribronchiolar environment (e.g. collagen fibers), contributing to a greater roundness of the bronchiole.

Chronic exposure to ozone induced changes in all morphometric parameters similar to those found after acute ozone exposure except for bronchiole and epithelial areas. The morphometric data after chronic ozone exposure concur with alterations of bronchial epithelium as obtained by descriptive visual analysis. However, the use of multiple morphometric parameters allowed a more reliable analysis of structural changes of bronchial epithelium than that performed by means of conventional semi-quantitative analysis of epithelial damage.

Comparison of morphometric parameters between acute and chronic ozone exposure groups emphasizes the importance of the duration of ozone exposure to the remodeling of small airway. Important differences were found for bronchial, lumen and epithelial areas, which are evidence of a more extensive remodeling of small bronchi after chronic ozone exposure.

In order to correlate the remodeling of bronchial epithelium with the expression of collagen in the lung, we conducted a quantification of peribronchial collagen in parallel to the assessment of morphometric parameters. Acute ozone exposure induced a significant increase of peribronchial collagen content similar to that observed under chronic ozone exposure. In addition, collagen content in the same lung part was also quantified by means of Sircol assay. However, acute exposure to ozone did not induce a significant increase of the total collagen content despite a significant increase in peribronchial collagen. We must emphasize that peribronchial collagen content represents a very small part of the total lung collagen which is distributed mainly around larger bronchi and vessels. Consequently, variations in the expression of peribronchial collagen did not impact on the global collagen content. The combined analysis of total collagen content in the lung with peribronchial collagen allows us to show that acute ozone exposure did not change the expression of collagen content in the entire lung lobe, but increased significantly the expression of peribronchial collagen. However, chronic ozone exposure induced a significant increase of collagen content both in the entire lung lobe and around small airways. These results indicate that the expression of peribronchial collagen is a very discriminative parameter for the assessment of small airway remodeling in addition to the epithelium structural changes.

Airways resistance is lower upon chronic ozone exposure as compared to a single exposure. Acute exposure causes severe injury of the respiratory epithelial barrier as previously shown^15^ that elicit a partial loss of epithelial layer and an increase of tight junctions followed by augmented pulmonary resistance. For chronic exposure, the tissue damage is more profound in the airways with emphysema resulting in pulmonary resistance, which is less than in the acute model.

The present study demonstrates that chronic ozone exposure, unlike acute ozone exposure, induced a significant increase of airspace density and airspace diameter (Feret diameter), but a significant decrease of the number of airspaces. Therefore, chronic ozone exposure induced, in addition to small airway remodeling, emphysema in agreement with literature data^14^. In the present study we used multiple and new morphometric parameters allowing a much more precise and reliable analysis compared to the standard mean linear intercept and chord length measurements (Lm), since the new morphometric parameters were applied on the entire lung section and without any manual intervention of the experimenter. It is worth noting that acute ozone exposure did not induce emphysema, which means that it did not affect significantly the structure of parenchymal tissue that is the alveolar septae, as indicated by the lack of effect on the expression of lung collagen content.

In conclusion, morphometric numerical analysis allows an automatic, unbiased and precise assessment of small airway remodeling. The structural alterations of the small airways correlated with functional changes enabling to follow the progression from acute to chronic ozone induced respiratory pathology with chronic inflammation, fibrosis and emphysema.

## Materials and Methods

### Animals

We used C57BL/6 female mice (Janvier Laboratory, France), 7–8 weeks old. Mice were bred and housed in specific pathogen-free animal facility at Transgenose Institute (TAMM-CNRS, UPS 44, Orleans, France; under agreement D-45-234-6, 2014). They were maintained in a temperature controlled (23 °C) facility with a strict 12 h light/dark cycles and free access to food and water. Animal husbandry and experimentation were conducted in accordance with relevant guidelines, regulations and approval of the French Institutional Committee under the agreement: CLE CCO 2015-1088.

### Ozone-induced airway inflammation

Mice were exposed to ozone in a plexiglas chamber (*EMB 104*, *EMMS*®) at 1ppm during 1 h for the acute model, and 1.5 ppm for 2 h, twice weekly during 6 weeks for the chronic model. For both models, analyses were performed 24 h after the last ozone exposure. Ozone is created by ozonisator (*Ozonisator Ozoniser S 500 mg*, *Sander®*) and levels was controlled by sensor (*ATI 2-wire transmitter*, *Analytical Technology®*). Mice were euthanized by progressive CO_2_ inhalation until 24 h after last ozone exposure and BAL was collected. After a cardiac perfusion with ISOTON II (*Acid free balanced electrolyte solution Beckman Coulter*, *Krefeld*, *Germany*) the lungs were collected and sampled for analyses.

### Flow cytometry and cytokine measurement

After ozone exposure, BAL was performed by four lavages of lung with 500 µL of saline solution (NaCl 0.9%) via a cannula introduced into the trachea of mice. BAL fluids were centrifuged at 2000 rpm for 10 min at 4 °C; the supernatants were stored at −20 °C for ELISA analysis and pellets were collected for FACS analysis.

TIMP-1 and MMP-9 in BAL supernatant were determined by ELISA (R&D systems, *Abingdon*, *UK*) following the manufacturer’s instructions. BAL cells were incubated with the antibodies for 25 minutes at 4 °C in FACS buffer (PBS, 2% FCS, 2 mM EDTA). The antibodies (BD Pharmingen) used are targeted against murine CD45 (553081) and EpCam (563478). Cells were washed with FACS buffer and fixed with 1 × lysing buffer (BD Pharmingen). To observe epithelial cells, we gated CD45^-^ then EpCam^+^ cells. Data were acquired using a flow cytometer (BD FacsCanto II) and analyzed with the FlowJo software (TreeStar, Mountain View, CA).

### Lung function measurements

Airway hyperresponsiveness was measured by using increasing concentrations of methacholine (25–200 mg/mL) using the FinePointe system (Buxco, DSI) as previously described^16^.

### Histopathology score

The left lobe of the lung tissue was fixed in 4% buffered formaldehyde and paraffin embedded under standard conditions. Tissue sections (3 µm) were stained with H&E and picrosirius red. Histological changes were determined by a semi-quantitative severity score (0 to 3) for inflammatory cell infiltration and alveolar epithelial injury (0 to 3) (Table 1). The slides were blindly examined by two independent investigators using a Nikon microscope (Nikon eclipse 80i, Country).

**Table 1:** Determination of scoring values for the semi-quantitative evaluation of inflammatory cell infiltration and small bronchi epithelial injury in acute and chronic ozone mouse models.

Score	Infiltration	Epithelial damage
0	No infiltration	No damage on epithelial cells
1	Little infiltration around vessels	Epithelial cells flattening
2	Little infiltration around vessels and bronchi	Complete epithelial cells flattening
3	High infiltration around vessels and bronchi	Desquamation of epithelial cells

### Automated histological image analysis

Lung slices were scanned at x20 magnification using a NanoZoomer-SQ and digital images of entire lung sections were recorded at x20, with a pixel size of 0.452 µm using the NDP.view.2 software (both from Hamamatsu Corporation, Hamamatsu, Japan). For the small airway remodeling study, we developed a numerical software program which enables, first, a fully automatic selection of small bronchioles, without smooth muscle layer, from the entire digital images of lung sections. The small bronchi were selected from their Feret diameter (the largest diameter) ranged between 100 and 500 µm. Secondly, it is processed to the automatically delineation of the external and internal limits of the bronchial epithelium. The external limit corresponds to the limit of the basal membrane and the internal limit to the lumen limit of the epithelial cells. Following these delineations multiple morphometric parameters of small bronchi were then assessed: (i) the bronchiole area, corresponding to the total area of the bronchiole from its external limit, (ii) the lumen area, corresponding to the total area from the internal limit of the bronchiole, (iii) the epithelium area, corresponding to the difference between the bronchiole area and the lumen area, (iv) the epithelium thickness, corresponding for each bronchiole to hundreds of measures performed all around the epithelium, (v) the circularity of the epithelium and the lumen, corresponding to the circularity of the external and internal limits of the bronchiole; the circularity index is a measure of the roundness of the bronchiole, is equal to 1 when the shape approximates a mathematically perfect circle (Fig. 1).

To characterize and quantify emphysema in entire lung sections, we developed a dedicated software program which allows a fully automatic assessment of key morphometric parameters of parenchymal tissue. Morphometric parameters were assessed from the same digital images used for the small airway remodeling analysis. Emphysema analysis was performed on digital images of entire lung sections in which we conducted a complete and automatic removal of all bronchi and vessels. After an automatic and accurate delineation of each airspace (alveoli and ducts) located in the entire lung section we proceeded to the assessment of the following morphometric parameters: (i) the airspace density, corresponding to the ratio of the area of airspaces versus the area of parenchymal tissue, (ii) the airspace diameter, corresponding to the Feret diameter (largest airspace diameter), and (iii) the number of airspaces per mm^2^ of parenchymal tissue.

### Quantification of collagen content

Collagen content was measured on the lung homogenate (same part of lung each time) supernatant, by the Sircol™ collagen assay (*biocolor*), following the manufacturer’s instructions. In addition, we developed a dedicated software program to automatically quantify the collagen content surrounding the small bronchi (peribronchial collagen). For this purpose, digital analysis was conducted on serial slides stained with picrosirius red. As described previously, it is first processed to automatically select small bronchi from digital images of entire lung sections, and subsequently proceeds to delineate the epithelium and peribronchial collagen. The expression of peribronchial collagen was expressed in percent and corresponds to the ratio of the area of peribronchial picrosirius red staining versus the sum of the area of the epithelium and peribronchial picrosirius red staining.

### Quantification of emphysema

To characterize and quantify emphysema in entire lung sections, we developed a dedicated software program which allows a fully automatic assessment of key morphometric parameters of parenchymal tissue. Morphometric parameters were assessed from the same digital images used for the small airway remodeling analysis. Emphysema analysis was performed on digital images of entire lung sections in which we conducted a complete and automatic removal of all bronchi and vessels. After an automatic and accurate delineation of each airspace (alveoli and ducts) located in the entire lung section we proceeded to the assessment of the following morphometric parameters: (i) the airspace density, corresponding to the ratio of the area of airspaces versus the area of parenchymal tissue, (ii) the airspace diameter, corresponding to the Feret diameter (largest airspace diameter), and (iii) the number of airspaces per mm^2^ of parenchymal tissue.

### Statistical analysis

Data are presented as mean ± standard error of mean. Statistical differences between control air group, acute and chronic exposure groups were analyzed by one-way analysis of variance (ANOVA) test with multiple Bonferroni’s comparison test for all parametric data or by Kruskal Wallis test for non-parametric data (GraphPad Prism 7.0; GraphPad Software, Inc. La Jolla, CA). A p-value < 0.05 was considered statistically significant.
